# Type I IFN autoantibodies underlie chikungunya live-attenuated vaccine encephalitis

**DOI:** 10.1073/pnas.2532212123

**Published:** 2026-01-22

**Authors:** Adrian Gervais, Paul Bastard, Qian Zhang, Marie-Christine Jaffar-Bandjee, Lucy Bizien, Lotfi Dahmane, Marie-Pierre Moiton, Julien Jabot, Radj Cally, Alexis Maillard, Etienne Frumence, Xavier de Lamballerie, Yazdan Yazdanpanah, Jérémie Rosain, Aurélie Cobat, Laurent Abel, Anne Puel, Cyril Ferdynus, Émilie Mosnier, Patrick Gérardin, Shen-Ying Zhang, Jean-Laurent Casanova

**Affiliations:** ^a^Laboratory of Human Genetics of Infectious Diseases, Necker Branch, INSERM U1163, Necker Hospital for Sick Children, Paris 75015, France; ^b^Imagine Institute, Paris Cité University, Paris 75015, France; ^c^St. Giles Laboratory of Human Genetics of Infectious Diseases, Rockefeller Branch, Rockefeller University, New York, NY 10065; ^d^Pediatric Hematology-Immunology and Rheumatology Unit, Necker Hospital for Sick Children, Assistance Publique-Hôpitaux de Paris, Paris 75015, France; ^e^Associated National Reference Center for Arboviruses, Centre Hospitalier Universitaire-Réunion, Saint-Denis, La Réunion 97400, France; ^f^Laboratoire de Microbiologie, Centre Hospitalier Universitaire de la Réunion-Site Nord, Saint-Denis, La Réunion 97400, France; ^g^Service de Maladies infectieuses, Centre Hospitalier Universitaire Félix Guyon, Saint-Denis, La Réunion 97400, France; ^h^Intensive Care Unit, Centre Hospitalier Universitaire, Saint-Denis, La Réunion 97400, France; ^i^Department of Intensive Care Medicine, Felix Guyon University Hospital, Saint-Denis, La Reunion 97400, France; ^j^Service de Réanimation médicale, Hôpital Saint-Louis, Assistance Publique Hôpitaux de Paris, Paris 75651, France; ^k^Unité des Virus Émergents (UVE: Aix-Marseille Univ, Università di Corsica, Institut de recherche pour le developpement 190, Inserm 1207, Institut de recherche biomédicale des armées), Marseille 13385, France; ^l^Department of Infectious Diseases, Bichat-Claude Bernard Hospital, Paris 75018, France; ^m^Study Center for Primary Immunodeficiencies, Assistance Publique Hôpitaux de Paris, Necker Hospital for Sick Children, Paris 75015, France; ^n^Centre for Clinical Investigation Clinical Epidemiology (INSERM CIC 1410), Centre Hospitalier Universitaire de La Réunion, Saint Pierre, La Reunion 97410, France; ^o^Service de Maladies Infectieuses et Tropicales, Centre Hospitalier Universitaire de La Réunion Sites Sud, Saint Pierre, La Réunion 97448, France; ^p^Processus Infectieux en Milieu Insulaire Tropical (UMR PIMIT), Université de La Réunion, CNRS 9192, Inserm 1187, IRD 249, Plateforme Technologique CYRO, Sainte-Clotilde, La Réunion 97490, France; ^q^HHMI, New York, NY 10065

**Keywords:** human autoantibodies, type I interferons, chikungunya vaccine encephalitis, live-attenuated vaccines

## Abstract

Human autoantibodies neutralizing type I interferons (IFNs) have emerged as strong, common, and global determinants of a growing number of severe viral diseases, including hypoxemic viral pneumonia, arboviral encephalitis, and adverse reaction to the live-attenuated yellow fever virus (YFV) vaccine. Chikungunya virus (CHIKV) is a growing global health concern that the live-attenuated vaccine VLA1553 (IXCHIQ®) was developed to address. In 2025, five unrelated adults (aged 82 to 88) on the island of La Réunion (France) developed severe reactions postvaccination; two died. The three patients with encephalitis (aged 84 to 85), including one lethal case, had immunoglobulin G autoantibodies in the blood neutralizing high concentrations of both IFN-α and -ω on admission. An 82-y-old survived rhabdomyolysis without encephalitis, and an 88-y-old died during hospitalization following CHIKV infection despite late vaccination; both lacked autoantibodies against type I IFNs. Autoantibodies neutralizing type I IFNs underlie all three cases of live-attenuated CHIKV vaccine encephalitis studied. Individuals with autoantibodies neutralizing type I IFNs should not be inoculated with live-attenuated YFV and CHIKV vaccines.

## Significance

Autoantibodies that block type I interferons (IFNs) have recently been recognized as major causes of a growing number of life-threatening viral infections. We report that these autoantibodies also explain severe brain inflammation in elderly individuals following vaccination with the live-attenuated chikungunya virus (CHIKV) vaccine, VLA1553 (IXCHIQ®). This finding reveals that type I IFN-neutralizing autoantibodies can cause severe adverse reactions to live-attenuated CHIKV vaccines, as they do for yellow-fever vaccines. Identifying individuals with these autoantibodies before vaccination could prevent such outcomes and improve vaccine safety for older adults.

Chikungunya virus (CHIKV) is a single-stranded RNA virus of genus *Alphavirus* within the *Togaviridae* family. It can be transmitted to humans by mosquitoes, triggering disease. CHIKV was first described in 1952 in Tanganyika, East Africa, where it is now endemic (1). CHIKV infection has become a significant public health concern globally, particularly in tropical and subtropical regions, where *Aedes* mosquitoes spread the virus. Up to 85% of people infected with CHIKV develop “chikungunya fever” (CHIKF), a rapid-onset illness characterized by high fever, asthenia, arthralgia, myalgia, headache, and rash, usually resolving within 1 wk (2). Less than 1% of infected individuals develop severe disease, such as encephalitis, myocarditis, hepatitis, and even multiple organ failure (3). About 1 to 5 individuals per 1,000 infected people die. No specific treatment against CHIKV infection is available (4). The first live-attenuated CHIKV vaccine (IXCHIQ®, VLA1553) licensed for general use was released in November 2023, following Food and Drug Administration (FDA) approval (Centers for Disease Control, CDC; DrugBank). This vaccine induces anti-CHIKV neutralizing antibodies in ~99% of recipients, with the maintenance of antibody levels 2 y postvaccination (5).

Data from clinical trials and postmarketing surveillance indicate that 10 to 30% of IXCHIQ® vaccine recipients experience mild to moderate adverse effects, including headache, fatigue, and myalgia (European Medicines Agency, EMA). A small subset of individuals (<3%) may display more severe or prolonged chikungunya-like adverse events (5–8). The risk of such events appears to be similar across age groups. In a phase III trial in 3,082 participants (aged 18 to 88 y, including 59 individuals over 65 y of age), two individuals (0.06%) were hospitalized for unexplained serious adverse events (SAEs): a 58-y-old woman for severe myalgia, and a 66-y-old man for atrial fibrillation and severe hyponatremia due to inappropriate antidiuretic hormone secretion (6). Following an outbreak due to a CHIKV strain that had diverged from the East/Central/South African lineage beginning in August 2024, a vaccination campaign targeting adults over the age of 65 y was implemented on the island of La Réunion in April 2025. This campaign was launched despite the alert issued in February 2025 by the Vaccine Adverse Event Reporting System following the occurrence of SAEs in 2024 in six individuals over the age of 65 y receiving the same vaccine in the United States (9). The vaccination campaign on La Réunion was rapidly halted after three individuals over the age of 80 y with comorbid conditions were hospitalized following SAEs (https://valneva.com/press-release/valneva-provides-update-on-recommendation-for-use-of-its-chikungunya-vaccine-by-french-authorities/?lang=fr). In 2021, we reported that preexisting immunoglobulin G (IgG) autoantibodies neutralizing type I interferons (AAN-I-IFN) could account for one third of rare, life-threatening reactions to the live-attenuated yellow fever vaccine (10). AAN-I-IFN are common determinants of a growing list of severe viral infections, including those triggered by several arboviruses (11, 12). They precede viral infection and are causal for severe disease (13, 14). We, thus, hypothesized that SAEs following administration of the CHIKV live-attenuated vaccine might also be caused by preexisting AAN-I-IFN.

## Methods

### Patient Recruitment.

We enrolled five patients with severe adverse reactions to the VLA1553 CHIKV vaccine from La Reunion in France. Sampling was performed during acute infection. Written informed consent for participation in this study was obtained from all participants and/or their legal guardians in accordance with local regulations. The full study protocol was approved by the French Ethics Committee “Comité de Protection des Personnes” the French National Agency for Medicine and Health Product Safety, the “Institut National de la Santé et de la Recherche Médicale,” in France (Protocol No. C18-41), and the Rockefeller University Institutional Review Board in New York (Protocol No. JCA-0700). The experiments for measurement of auto-Abs to type I IFNs were conducted in France and the United States of America, in accordance with local regulations and guidance from the French National Agency for Medicine and Health Product Safety, the Institut National de la Santé et de la Recherche Médicale in Paris, France, and with the approval of the IRB of the Rockefeller University in New York, NY, respectively.

### Detection of Autoantibodies Against Type I-IFNs in Neutralization Assays and ELISA.

The HEK293T cell-based luciferase reporter assay was performed as previously described (15). The A549 cell-based luciferase reporter assay was performed as previously described (16). The detection of auto-Abs against IFN-I by ELISA was performed as previously described (17).

## Results

### Five patients with severe adverse reactions to the VLA1553 CHIKV vaccine.

We studied the five unrelated patients presenting SAEs following vaccination in April 2025 with the live-attenuated CHIKV vaccine (VLA1553/IXCHIQ®, Table 1) on La Réunion, a French island in the Indian Ocean. All patients were hospitalized for 2 to 6 d following vaccination. P1 was an 84-y-old man hospitalized for encephalitis and acute renal failure post vaccination; he died 14 d after vaccination (18). The CHIKV vaccine strain was detected in his blood and cerebrospinal fluid (CSF) by PCR. P2 was an 84-y-old man who developed CHIKF-like symptoms 48 h postvaccination, with an altered mental state and other symptoms of encephalitis 4 d after vaccination. He was hospitalized for 1 mo and then discharged home with cognitive impairment. The CHIKV vaccine strain was detected in his blood on admission; the CSF sample tested negative, but lumbar puncture was not performed until 10 d after admission. P3 was an 82-y-old man presenting with fever, worsening joint pain, and malaise 3 d after vaccination, necessitating hospitalization for 11 d; he was then discharged home. The CHIKV vaccine strain was detected in his blood; no lumbar puncture was performed. P4 was an 85-y-old man with fever, fatigue, and symptoms of encephalitis/encephalopathy resulting in multiple falls 4 d post vaccination. He was hospitalized for 10 d and recovered. Finally, P5 was an 88-y-old man previously diagnosed with dementia who had been naturally infected with CHIKV 3 wk earlier, which worsened his overall status. He was hospitalized 2 wk after infection, was vaccinated against CHIKV 5 d later, and died 6 d postvaccination. All five patients had a history of other medical conditions, but not of severe infections (Table 1). The vaccine was administered to 6,418 adults aged 18 y or older on La Réunion, including ~4,500 individuals aged 65 and older, and ~1,000 individuals over the age of 80 y.

**Table 1.** Clinical information of the five patients

Patient	P1	P2	P3	P4	P5
Sex (M/F)	M	M	M	M	M
Age (yrs)	84	84	82	85	88
Time from vaccination to symptoms onset (days)	3	2	4	4	N.A^*^
Symptoms at onset	Fever, Fatigue, Asthenia, Polyarthralgia, fall, diffuse arthralgia, confusion.	Fever, Fatigue, distal arthralgie, confusion.	Fever, worsening joint pain, fatigue, fall.	Fever, fatigue, multiple fall.	Continuous decline in general condition.
Disease progression	Confirmed encephalitis (IEC definition^†^). Renal failure. Deceased ~2 wk post vaccination.	Possible encephalitis (IEC definition). Discharged home with partial recovery, cognitive disorders in executive functions.	Possible respiratory system failure. Altered mental status. Discharged home with full recovery, after 11 d of hospitalization.	Possible encephalitis (IEC definition). Thrombopenia. Lymphopenia. Rhabdomyolysis. Discharged home with full recovery, after 10 of days hospitalization.	Weakened respiratory system function. Deceased 6 d post vaccination. Weakened respiratory system function.
ICU	Yes	No	No	No	No
CHIKV PCR positive	Blood, CSF	Blood^‡^	Blood	Blood	Blood
Post-vaccination delay to PCR positive (days)	7	10	5	7	4
CHIKV strain	Vaccine strain	Vaccine strain	Vaccine strain	Vaccine strain	Vaccine strain
Previous infectious disease history	Unremarkable	Unremarkable	Reiter’s disease (1980)	2024: one episode of cellulitis. Chronic wound on left ankle. Nov 2024: thoracic herpes zoster.	Nov 2024: Herpes zoster, post-herpetic neuralgia. Dec 2024: pneumonia. Jan 2025: viral bronchiolitis. 3 wk before vaccine: CHIKV infection.
Other previous medical conditions	Type 2 diabetes. Asthma. Ischemic heart disease (2005). Obstructive sleep apnea. Hiatal hernia. Hypercholesterolemia. Bilateral cataract.	Type 2 diabetes. Chronic kidney disease. Hearing loss. Gout.	HLA-B*27+ Ankylosing spondylitis. Bilateral cataracts. Obstructive sleep apnea. Benign paroxysmal positional vertigo. Deep vein thrombosis (2008). Pulmonary embolism (2012). Lumbar spinal stenosis. Knee arthroplasty. Benign prostate. hyperplasia. Immunosuppressive context.	Type 2 diabetes. Asthma. Minor thrombopenia. Obstructive sleep apnea. Arterial hypert ension.	Neurological and cardiovascular conditions. Neuropsychiatric and cognitive disorders. Ophthalmologic and ENT History. Other Medical History^§^.

^*^The patient was hospitalized on April 14, due to progressive deterioration of general health following natural CHIKV infection about 2 wk earlier. The patient subsequently received the live-attenuated CHIKV vaccine (IXCHIQ®/VLA1553) on April 19, 2025. The patient’s general condition further declined, and died 6 d post vaccination.

^†^IEC: International Encephalitis Consortium. The IEC criteria for confirmed and possible encephalitis are described in DOI: 10.1093/cid/cit458.

^‡^CHIKV PCR was also performed in CSF taken 10 d after disease onset, and revealed negative result but positive IgM and negative IgG.

^§^The patient had various previous medical conditions, including 1) neurological and cardiovascular history: Ischemic stroke (vertebrobasilar territory) in September 2023 with residual left hemiparesis; Troponin elevation without symptoms led to diagnostic coronary angiography on 29/09/2023, showing Hypertension and orthostatic hypotension (Eupressyl + Bipreterax) with iatrogenic hyponatremia (thiazide-related), Dyslipidemia, and Atrial fibrillation not mentioned explicitly but should be verified in context of stroke. Neuropsychiatric and Cognitive Disorders, with presentations of Severe mixed dementia (vascular and Alzheimer’s disease), diagnosed in 2019, Major depressive episode with apathy, behavioral disturbances, and sleep disorders with nightmares, Traumatic subdural hematoma in 2019 with persistent cognitive impairment, Traumatic brain injury in 2018 (bike vs. car accident) with probable loss of consciousness, rib fractures, pulmonary contusions, and subdural hematoma (no surgical indication), Delirium due to medication overdose (tramadol + morphine), requiring ICU stay (24 h). 2) Ophthalmologic and ENT History: Glaucoma, right eye, treated. Bilateral ectropion repair via lateral canthoplasty under local anesthesia (02/2022). Right upper eyelid ptosis repair (08/2017). Divergent strabismus surgery, right eye (06/2016). Bilateral cataract surgery under topical anesthesia. 3) Other Medical History: Osteoporotic fracture, with L3 vertebral compression fracture treated with cementoplasty. Sigmoid diverticulosis, Untreated obstructive sleep apnea, Asthma.

### AAN-I-IFN in three patients with CHIKV vaccine encephalitis.

**Fig. 1.**
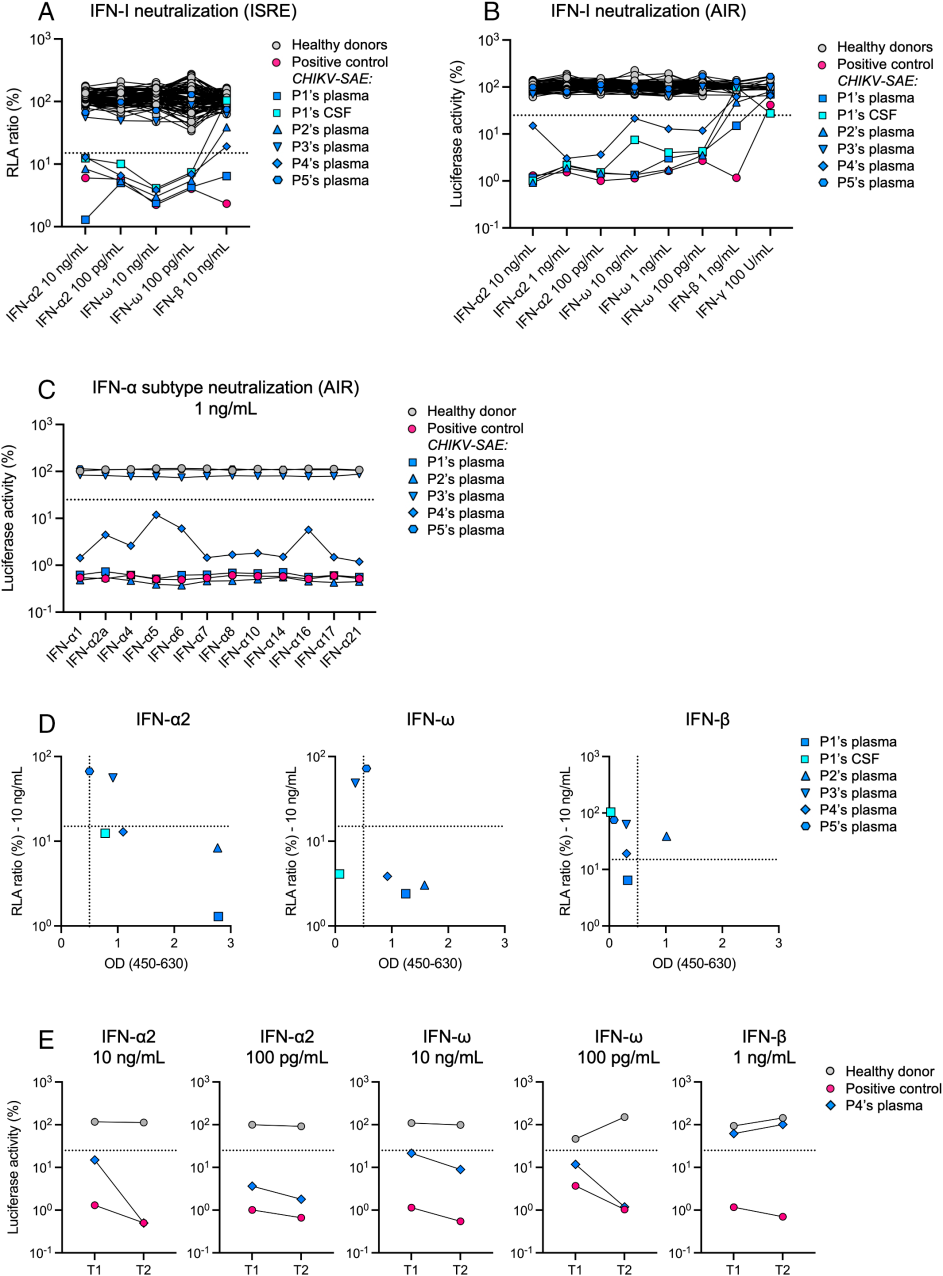
Evaluation of AAN-I-IFN in five patients with severe adverse events to the CHIKV vaccine. (*A*) Luciferase-based neutralization assay in HEK293T cells to detect autoantibodies neutralizing 10 ng/mL or 100 pg/mL of IFN-α2 or IFN-ω, or 10 ng/mL of IFN-β. Plasma and CSF samples from the five patients were diluted 1:10. Plasma from a patient with APS-1 previously confirmed positive for anti-IFN autoantibodies was included as a positive control. A Relative Luciferase Activity (RLA) <15% of the value for the median value of Luciferase activity of healthy controls was considered to correspond to neutralizing activity [dotted line, (15)]. This cutoff value was previously determined by testing a large number of healthy controls. (*B*) Luciferase-based neutralization assay with AIR (A549 interferon-responsive) cells to detect autoantibodies neutralizing 10, 1 ng/mL or 100 pg/mL of IFN-α2 or IFN-ω, or 1 ng/mL IFN-β, or 100 U/mL IFN-γ. Plasma and CSF samples from the five patients were diluted 1:20. A Luciferase Activity <25% of the value for the median value of Luciferase activity of healthy controls was considered to correspond to neutralizing activity [dotted line, (16)]. This cutoff value was previously determined by testing a large number of healthy controls. (*C*) Luciferase-based neutralization assay in AIR cells to detect autoantibodies neutralizing 1 ng/mL of each IFN-α subtype. Plasma samples were diluted 1:20. (*D*) Correlation between the structural detection of anti–IFN-I autoantibodies by ELISA and the neutralizing activity of the corresponding plasma or CSF samples. Neutralization data were obtained with HEK293T cells and 10 ng/mL IFN-I. (*E*) Luciferase-based neutralization assay in AIR cells to detect AAN-I-IFN in a longitudinal sample (T2) from Patient 4 collected 5 wk after the initial sample (T1).

We first tested the plasma of all five patients and the CSF of P1 for the presence of AAN-I-IFN, using a HEK293T cell-based luciferase reporter assay (15). Strikingly, we found that P1, P2, and P4, the three patients with postvaccine encephalitis, all had auto-Abs neutralizing 10 ng/mL of both IFN-α2 and IFN-ω. This is the highest level of neutralization that can be detected, attesting to the ability of the patients’ blood to neutralize at least 100 ng/mL (concentrations of type I IFNs in the pg/mL range can normally activate cells). P1 also had auto-Abs neutralizing 10 ng/mL IFN-β. Such auto-Abs were also detected in the CSF of P1 and neutralized high concentrations of both IFN-α2 and IFN-ω (Fig. 1*A*). These results were confirmed by the detection of auto-Abs neutralizing the same IFN-I in a different A549 cell-based luciferase reporter system (16) (Fig. 1*B*). As previously reported (15, 19), the IFN-α2-neutralizing plasma samples from P1, P2, and P4 neutralized all 12 subtypes of IFN-α (Fig. 1*C* and *SI Appendix*, Table S1). The IgG auto-Abs responsible for this neutralization activity were also detected by ELISA in plasma from these three patients (Fig. 1*D*). They were also found in the only longitudinal sample available from P4, collected 5 wk after the initial sample (Fig. 1*E*). By contrast, they were not detected by neutralization assays nor by ELISA in samples from P3 (an 82-y-old who survived rhabdomyolysis without encephalitis) and P5 (an 88-y-old who died during hospitalization following CHIKV infection despite late vaccination) (Fig. 1 *A* and *B* and *SI Appendix*, Table S1). IgG auto-Abs neutralizing high concentrations of all 12 IFN-α and IFN-ω were found in all three patients with postvaccine encephalitis, whereas these auto-Abs were not detected in the two patients without encephalitis, including the patient who experienced natural CHIKV infection before vaccination.

## Discussion

We report that three of five patients (60%) with severe disease triggered by live-attenuated VLA1553 CHIKV had blood AAN-I-IFN on admission. Their samples neutralized all 12 IFN-α subtypes and IFN-ω at high concentrations (10 ng/mL, blood diluted 1/20); one also neutralized IFN-β. These three patients, aged 84 or 85, all had encephalitis. One died and had auto-Abs against IFN-α2, IFN-β, and IFN-ω. All had high levels of AAN-I-IFN in blood. The presence of blood-circulating AAN-I-IFN impaired antiviral I-IFN immunity, resulting in replication of the live-attenuated virus, as it has been shown previously for other viruses (10, 19–21), including live-attenuated Yellow Fever virus vaccine (10), thereby precipitating viral encephalitis.

AAN-I-IFN neutralizing low (100 pg/mL) or high (10 ng/mL) concentrations of type I IFNs are present in ~0.5% of the general population under 65, and ~5% over age 65 (14, 15). The prevalence of AAN-I-IFN neutralizing IFN-α and IFN-ω at 10 ng/mL is 0.40% (95% CI: 0.28 to 0.52%) and 1.21% (95% CI: 0.75 to 1.96%) in individuals aged over 65 or 80 to 85, respectively, in the general population (15). Compared with this group, individuals with these autoantibodies had a Firth-corrected odds ratio of 110.6 (95% CI: 20.2 to 704.0) for CHIKV vaccine SAEs, and 553 (95% CI: 50.8 to 75,503.7) for encephalitis. A limitation to consider is that only a small number of patients were studied, and all the patients lived in the La Reunion island. As ~4,500 and ~1,000 people over 65 or 80, respectively, were vaccinated on La Réunion, we can estimate that ~10 to 20 over 65 and ~5 to 10 over 80, perhaps had similar AAN-I-IFN levels, suggesting that these antibodies may underlie SAEs with incomplete penetrance. Age may be another risk factor predisposing to life-threatening SAEs, including encephalitis. AA-I-IFN may also contribute to adverse reactions in individuals under 65 or 80 y of age, however no related clinical data are available currently for an overall evaluation. Further studies across age groups are now required to confirm these findings.

These findings align with our prior report that one-third of SAEs from the live-attenuated YFV vaccine are due to AAN-I-IFN neutralizing high levels of IFN-α and IFN-ω (10). Historically, YFV vaccination was gradually contraindicated in the elderly due to SAEs, probably caused by AAN-I-IFN (22). AAN-I-IFN are also common (with a prevalence ranges from ~0.5 to ~5% in the young and elderly), strong (with OR of ~10 to over 500 for severe viral infection predisposition), and global determinants of a growing number of severe infections with wild-type viruses, including respiratory viruses (SARS-CoV-2, MERS, Influenza virus), and arboviruses (West Nile, tick-borne encephalitis, Powassan, Usutu, and Ross River viruses) (10, 13–15, 19, 20, 23–73). The predisposition of patients with AAN-I-IFN to severe disease caused by the YFV and CHIKV vaccines suggests that these individuals may also be prone to severe disease triggered by wild-type YFV or CHIKV infection, such as encephalitis (74). Testing patients with severe YFV or CHIKV disease for AAN-I-IFN is therefore warranted.

These findings have key clinical implications. First, individuals with AAN-I-IFN should not receive the VLA1553 (75). Second, those with adverse reactions to VLA1553 should be tested for AAN-I-IFN. Third, affected patients may benefit from early treatment with recombinant IFN-β, if they lack auto-Abs against it. Fourth, individuals over 65, or younger people with autoimmune conditions or a history suggesting increased viral susceptibility, should be screened before vaccination with VLA1533. Finally, although challenging in some areas, AAN-I-IFN screening could be considered for all age groups prior to VLA1553 vaccination. A recently developed whole blood-based assay can assess the presence of AAN-I-IFN in a faster and simpler manner (76). It should be considered for general application in clinical laboratories. Inactivated viruses, recombinant proteins, or mRNA vaccines are safer alternatives for individuals with AAN-I-IFN, who display normal Ab responses to mRNAs and proteins (14, 77).

## Supplementary Material

Appendix 01 (PDF)

## Data Availability

Study data are included in the article and/or *SI Appendix*.
