# Supplementary material for: Type I IFN autoantibodies underlie chikungunya live-attenuated vaccine encephalitis
Source: Proc Natl Acad Sci U S A. 2026 Jan 22;123(4):e2532212123. doi: 10.1073/pnas.2532212123 (PMC12846812; doi:10.1073/pnas.2532212123)
Supplement: Supplementary file 1 — Appendix 01 (PDF) [file pnas.2532212123.sapp.pdf]

Supplementary Information for

Type I IFN autoantibodies underlie chikungunya live-attenuated vaccine encephalitis

Adrian Gervais, Paul Bastard, Qian Zhang, Marie-Christine Jaffar-Bandjee, Lucy Bizien, Lotfi Dahmane, Marie-Pierre Moiton, Julien Jabot, Radj Cally, Alexis Maillard, Etienne Frumence, Xavier de Lamballerie, Yazdan Yazdanpanah, Jérémie Rosain, Aurélie Cobat, Laurent Abel, Anne Puel, Cyril Bruno Ferdynus, Émilie Mosnier[$], Patrick Gérardin[$], Shen-Ying Zhang[§,@], Jean-Laurent Casanova[§,@]

[$],[§]: Equal contributions
[@]: Correspondence: Shen-Ying Zhang (shzh289@rockefeller.edu) and Jean-Laurent Casanova (casanova@rockefeller.edu)

This PDF file includes:

- Supplementary Table 1

Supplementary Table 1. Evaluation of autoantibodies against type I interferons (AAN-I-IFN) in five patients with severe adverse events to the CHIKV live-attenuated vaccine

Patient	IFN-I neutralized (10 ng/mL)	IFN-α subtypes neutralized (1ng/mL)
P1	IFN-α2, IFN-β, IFN-ω	IFN-α1, IFN-α2a, IFN-α4, IFN-α5, IFN-α6, IFN-α7, IFN-α8 IFN-α10, IFN-α14, IFN-α16, IFN-α17, IFN-α21
P2	IFN-α2, IFN-ω	IFN-α1, IFN-α2a, IFN-α4, IFN-α5, IFN-α6, IFN-α7, IFN-α8, IFN-α10, IFN-α14, IFN-α16, IFN-α17, IFN-α21
P3	none	none
P4	IFN-α2, IFN-ω	IFN-α1, IFN-α2a, IFN-α4, IFN-α5, IFN-α6, IFN-α7, IFN-α8, IFN-α10, IFN-α14, IFN-α16, IFN-α17, IFN-α21
P5	none	none

Note: the neutralization results for the highest concentrations of type I IFNs tested are displayed.
